# Sintering Behavior and Mechanical Properties of Mullite Fibers/Hydroxyapatite Ceramic

**DOI:** 10.3390/ma11101859

**Published:** 2018-09-29

**Authors:** Xueni Zhao, Qingyao Liu, Jianjun Yang, Weigang Zhang, Yao Wang

**Affiliations:** College of Mechanical and Electrical Engineering, Shaanxi University of Science and Technology, Xi’an 710021, China; 18840243585@163.com (Q.L.); 18309293644@163.com (J.Y.); zhangwg0103@163.com (W.Z.); wyyao1199@163.com (Y.W.)

**Keywords:** hydroxyapatite, mullite fiber, sintering, densification mechanism

## Abstract

The effect of fiber content and sintering temperature on sintering behavior and mechanical properties of mullite fibers/hydroxyapatite composites was studied. The composites were fabricated by hydrothermal synthesis and pressureless sintering. The amount of fibers was varied from 5 wt % to 15 wt % through hydrothermal synthesis, mullite fibers and hydroxyapatite composite powders were subsequently sintered at temperatures of 1150, 1250, and 1350 °C. The composites presented a more perturbed structure by increasing fiber content. Moreover, the composites experienced pore coalescence and exhibited a dense microstructure at elevated temperature. X-ray diffraction indicated that the composites underwent various chemical reactions and generated silicate glasses. The generation of silicate glasses increased the driving force of particle rearrangement and decreased the number of pores, which promoted densification of the composites. Densification typically leads to increased hardness and bending strength. The study proposes a densification mechanism and opens new insights into the sintering properties of these materials.

## 1. Introduction

Calcium phosphates have aroused great interest in interdisciplinary fields of sciences involving chemistry, biology, and medicine. These compounds are widely used as biomaterials because they can induce cell osteoblast differentiation [1,2]. Hydroxyapatite (HA, Ca_10_(PO_4_)_6_(OH)_2_) is the most thermodynamically stable calcium phosphate salt, and has been extensively studied because it is a major mineral component of human hard tissue and vertebrate skeletal systems [3,4]. After it is implanted into the human body, calcium and phosphate ions are separated from HA and absorbed by the body, which helps the growth of new bone tissues [5]. From a mechanical point of view, HA has an excellent biological nature including osteoconductivity, biocompatibility, and bioactivity, but the wider use of HA in biomaterials is highly restricted due to its high brittleness and poor fracture toughness [6]. Under the above backdrop, HA-based composites have been explored by introducing different reinforcements to improve the mechanical properties of HA, in order to make HA more relevant in the biomedical engineering and applications [7,8]. So far, the widely studied HA-based composites include HA-alumina [9,10], HA-zirconia [11], and HA-bioglass [12,13] composites.

Mullite (Al_4+2x_Si_2−2x_O_10−x_), one of the most important ceramic materials, is the only stable intermediate phase in alumina-silica systems at normal atmospheric pressure. It is also a solid solution phase of alumina and silica that is commonly found in synthetic ceramic, and rarely exists as a natural mineral [14]. Due to its high flexural and compressive strength, many of its properties are superior to other metal oxides at elevated temperature [15,16]. In addition, mullite fiber (MF) is biologically inert, low-cost, remains stable after being implanted in the human body, and causes no rejection. It is considered as one of the most hopeful candidate materials for reinforcing ceramic materials. There have been some limited studies and applications of mullite-reinforced HA composites in recent years. Dubey et al. [17] used spark plasma sintering to obtain mullite–HA composites. The results revealed that densification and a high hardness exceeding 7 GPa were achievable at the lower temperature. Ebadzadeh et al. [18] prepared porous ceramics through the reaction sintering of clay, alumina, and HA. The mullite content of samples was increased from 5 wt % to 10 wt % at 1350 °C, and the porosity increased from 6.8% to 21.3%. Yetmez et al. [19] considered sintering effects on the properties of bovine-derived HA doped with powder mullite. With sintering increasing temperature from 1000 to 1300 °C, the Vickers hardness, density, and compressive strength reached 1369 HV, 2.62 g/cm^3^, and 101 MPa, respectively. Nath et al. researched the combined effect of mullite addition and sintering conditions on the properties of calcium phosphate–mullite composites. The results revealed that close to 95% theoretical density was achieved by sintering in the temperature range of 1300–1350 °C. The composites could achieve better mechanical properties, such as fracture toughness, compressive, and flexural strength [20,21]. To date, however, there have been few studies exploring the influence of sintering reaction on the densification mechanism and mechanical properties of the composites. Therefore, further study of the effect of MF on sintering behavior and mechanical properties of mullite fibers/hydroxyapatite (MF/HA) composites is needed. Moreover, the dispersion of fibers in a matrix is one of the most critical problems for composites, which is a prerequisite for their applications as additives for reinforcement of the composites. If fibers are unevenly dispersed in matrix, the composites tend to crack at fiber-lean regions under low stresses. Fiber agglomeration occurs in fiber-rich regions. During the preparation of MF-reinforced HA composites, micron-scale fibers are apt to agglomerate.

With this background, very pure and crystalline HA powders were prepared by hydrothermal synthesis method. At the same time, MF was introduced in the hydrothermal process to further improve the dispersion of fibers in the HA matrix, which enabled the achievement of homogeneous nucleation and growth of HA on the surface of MF and in the gaps among fibers. Thereafter, pressureless sintering was used to prepare MF/HA composites at 1150–1350 °C. The present paper describes microstructure evolution and phase assemblages of MF/HA composites as a function of fiber content and sintering temperature. The composites were analyzed by detailed thermal analysis X-ray diffraction (XRD), scanning electron microscope (SEM), Fourier transform infrared spectroscopy (FTIR), and thermogravimetry–differential scanning calorimetry (TG–DSC) to develop a qualitative understanding of sintering behavior and mechanisms of MF/HA composites. Based on microstructure, phase composition, and detailed thermal analysis, the macroscopic mechanical properties of MF/HA composites were further explored. A possible densification process of the composites is also described at the end of the paper. According to the effects of fiber content and sintering temperature on the sintering behavior and mechanical properties of MF/HA composites, this study also clarifies the sintering properties of these materials.

## 2. Materials and Methods

### 2.1. Materials

Different amounts of mullite fibers (99% pure, with an average diameter of 6.7 μm and length of 30–40 mm, from Qinxing high temperature fiber Co., Deqing, China) were added into HA powders as reinforcing agent. Calcium nitrate tetrahydrate (Ca(NO_3_)_2_*·*4H_2_O), di-ammonium hydrogen phosphate ((NH_4_)_2_HPO_4_), ammonia water (NH_4_OH), and anhydrous ethanol (C_2_H_6_O) were supplied by Sinopharm Chemical Reagent Co., Ltd. (Shanghai, China). All of the chemical reagents were of analytical grade and used as received without further purification. Deionized water was used for rinsing and makeup of all aqueous solutions throughout the study.

### 2.2. Methods

**Figure 1:**
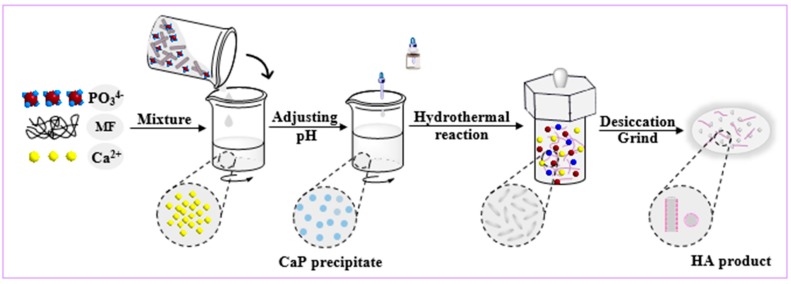
Schematic of processing strategies for the fabrication of mullite fiber (MF)/hydroxyapatite (HA) composite powders via hydrothermal method.

HA was fabricated by a hydrothermal synthesis method. In a typical experiment, 0.167 mol/L Ca(NO_3_)_2_ solution and 0.100 mol/L (NH_4_)_2_HPO_4_ solution were obtained by dissolving Ca(NO_3_)_2_*·*4H_2_O and (NH_4_)_2_HPO_4_ in deionized water, respectively. The MF was immersed in (NH_4_)_2_HPO_4_ solution, and mass fractions of fibers were set at 5 wt %, 10 wt %, and 15 wt %. (NH_4_)_2_HPO_4_ solution with quantitative fibers was added dropwise within 10 min into Ca(NO_3_)_2_ solution with magnetic stirring. The pH of mixed solutions was adjusted to 10.0 by adding ammonia solution at room temperature, and then they were continuously stirred for 15 min. After that, the obtained mixed solution was transferred to a Teflon-lined stainless-steel autoclave with 300 mL capacity. Then, the autoclaves underwent hydrothermal reaction at 170 °C for 24 h. Subsequently, the autoclave was cooled down naturally and the precipitate was washed with deionized water and anhydrous ethanol three separate times. Finally, the remaining aqueous dispersion was dried for 3 h at 80 °C, and was then ground into powder to prepare MF/HA composite powders. The composite powders were pressed into a circular sheet (Ф 10 mm × 2 mm) and strips (40 mm × 18 mm × 4 mm) of green body with a uniaxial cold press at 8 MPa. All the green bodies were sintered at 1150–1350 °C for 20 min. The heating rate was 10 °C/min under 500 °C and maintained at that level for 30 min, then raised to 1150–1350 °C with a rate of 5 °C/min rate and maintained at that level for 20 min. The manufacturing process was performed as shown in Figure 1.

### 2.3. Characterization

The phase compositions of the composites were examined by X-ray diffraction (XRD; AD/max2200/PC, Rigaku, Japan) operated at 40 kV with a 40 mA Cu Kα radiation source. The morphological characteristics and microstructure of MF and the composites were investigated using a JEOL JSM-6460 scanning electron microscope (SEM). The powder of the sintered sample was examined by Fourier transform infrared spectroscopy (FTIR, Vertex 70, Bruker Corporation, Germany) to identify different chemical groups in the composites. The thermal stability of composites was tested by thermogravimetry (TG) and differential scanning calorimetry (DSC) tests using a STA409PC Synchronous Integrated Thermal Analyzer, operated under nitrogen atmosphere from room temperature to 1300 °C with a heating rate of 10 °C/min. The bulk density of composites was determined by Archimedes’ method using deionized water as liquid medium. Vickers hardness (HV) of polished sintered samples was examined by a Vickers hardness tester (HX-1000TM/LCD, Shanghai, China). For this purpose, a 1 kg load was applied on the polished sample with a dwell time of 15 s to produce indentation. Three measurements were taken at different locations of the samples to obtain the average value. Three-point bending tests were conducted with a loading rate of 0.05 mm/min and 20 mm span to acquire the bending strength of the samples by universal 1036PC testing machine. For each experiment, at least three specimens were tested. The bending strength was calculated by following Equation [22]:

*σ_f_* = 3*FL*/(2*bh*^2^) (1)

where *F* is fracture force (N), *L* is the span length (mm), *b* is sample width (mm), h is the sample height (mm), and *σ_f_* is the three-point bending strength (MPa).

## 3. Results and Discussion

### 3.1. Morphological Analysis

**Figure 2:**
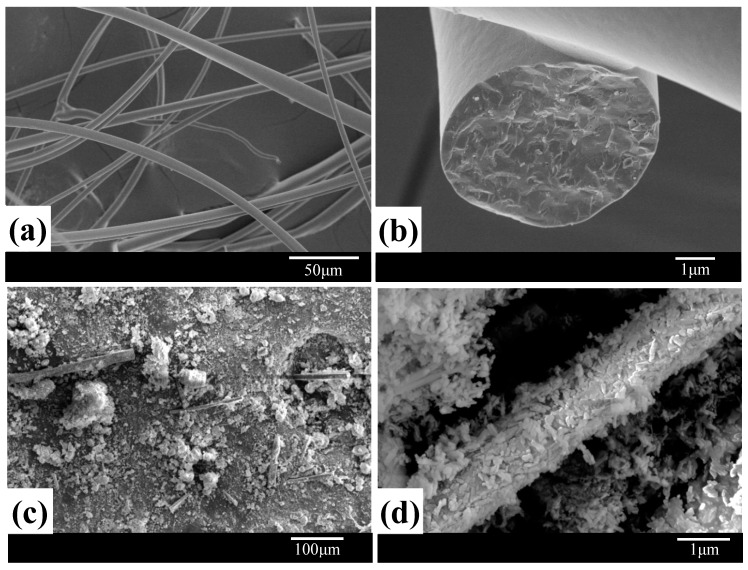
The morphology of mullite fibers: (**a**,**b**) untreated; (**c**,**d**) after hydrothermal treatment.

The SEM images of MF are shown in Figure 2 without (a, b) and with (c, d) hydrothermal treatment. The MF surfaces were smooth before hydrothermal treatment. However, highly homogeneous and dense HA crystals formed on the fiber surfaces. HA crystals deposited on the fiber surface filled the gaps between fibers and matrix, which could provide a good interface for the combination of fibers and matrix in the subsequent sintering process. In addition, the nucleation and growth of HA crystals on the surface of fibers and in the gap between fibers improved the dispersion of MF in the matrix. This is because HA crystals avoid direct contact with neighboring fibers to alleviate partial agglomeration of fibers. Therefore, MF/HA composite powders were fabricated by hydrothermal synthesis for subsequent studies.

**Figure 3:**
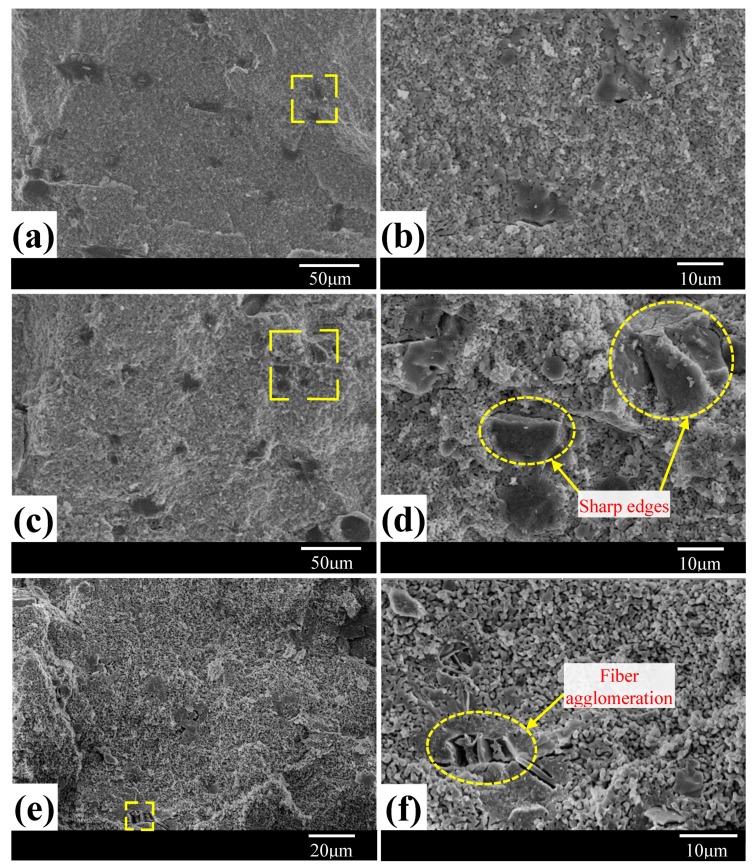
SEM images of MF/HA composites with various MF contents at 1150 °C sintering: (**a**,**b**) 5 wt %; (**c**,**d**) 10 wt %; and (**e**,**f**) 15 wt %.

Fibers should be evenly dispersed to achieve effective binding to HA particles. If MF can be evenly dispersed in matrix, the property of the composites would be significantly improved. In order to clarify the changes in morphology of MF/HA composites as a function of fiber content and sintering temperature, SEM analysis of the samples was conducted. Figure 3 presents the morphological changes occurring in samples sintered at 1150 °C with various MF contents (5–15 wt %). When fiber content was low, fibers could be uniformly dispersed in the HA matrix. However, with increasing fiber content, the dispersion state of fibers changed and further increased MF content (15 wt %) caused partial agglomeration of fibers, as depicted in Figure 3f. This is related to the dispersion state of MF during hydrothermal treatment. In addition, a more perturbed structure was found with increased MF content. The observation is owing to the formation of new phases. The uniformity of the micromorphology decreased with increasing MF content after sintering at elevated temperature.

**Figure 4:**
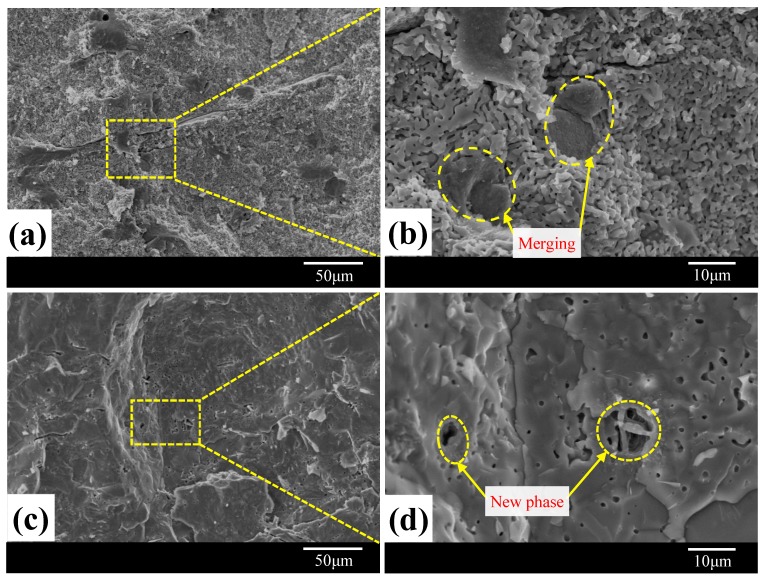
SEM images of MF/HA composites after sintering at different temperatures with 10 wt % MF content: (**a**,**b**) 1250 °C; (**c**,**d**) 1350 °C.

Figure 4 shows the micromorphological evolution of 10 wt % MF-containing samples versus sintering temperatures at 1250–1350 °C. The porous microstructure of MF/HA composites sintered at 1150 °C is presented in Figure 3c,d. No significant grain growth was observed and almost all of the spherical grains were fine. There were still large gaps between fibers and matrix. As sintering temperature increased to 1250 °C, a significant change of microstructure occurred (Figure 4a,b) and grain growth was accelerated, resulting in the formation of a widespread vermicular microstructure and tiny aggregates of nearly equiaxed grains. Simultaneously, the bonding between fiber and matrix gradually tightened, which is ascribed to the ability of MF to react with CaO decomposed by HA at high temperature [20]. When the composites were sintered at 1350 °C, the microstructure of the composites (Figure 4c,d) experienced pore coalescence and exhibited a dense microstructure. Additionally, it is clear that the higher temperature sintering led to the merging and enlargement of grains, which is probably due to the diffusion effects and generation of new phases at grain boundaries. Compared to the composites prepared at 1150 °C and 1250 °C, pores were presented (Figure 4d) with varying size and distribution. Irregularly shaped pores having a size in the range of 1–2 μm were observed. The origin of the pore can be explained by the difficulty in sintering with the addition of MF and the generation of gas due to the decomposition reaction. This will be discussed in a later section. Herein, when the sintering temperature was raised from 1150 °C to 1350 °C, the micromorphology of MF/HA composites changed drastically due to chemical reaction (as described in Section 3.2) being able to proceed to completion.

### 3.2. Sintered Phases

**Figure 5:**
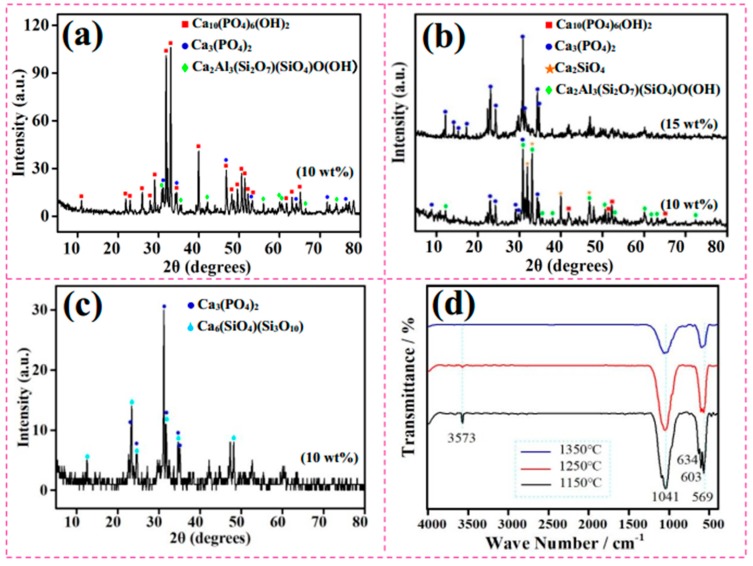
XRD spectra of MF/HA composites sintered at: (**a**) 1150 °C—10 wt %; (**b**) 1250 °C—10/15 wt %; (**c**) 1350 °C—10 wt %; and (**d**) Fourier transform infrared (FTIR) spectra of the composites with the addition of 10 wt % MF.

To verify the crystalline structures of MF/HA composites synthesized via hydrothermal method and pressureless sintering, XRD and FTIR studies were carried out. Figure 5 displays the phase evolution in the composites. Figure 5a displays the XRD pattern of the sample sintered at 1150 °C with 10 wt % MF. It exhibits three characteristic high-intensity diffraction peaks for HA at 2θ values of about 31.902°, 32.320°, and 33.040°, which is very consistent with the reference data of JCPDS 9-432. In particular, the strongest intensity peak corresponding to the HA diffraction peak was found at 32.320°, which is a strong evidence of HA crystallinity. In the meantime, two additional phases were also detected in the XRD pattern: zoisite (Ca_2_Al_3_(Si_2_O_7_)(SiO_4_)O(OH)) and tricalcium phosphate (TCP, Ca_3_(PO_4_)_2_). This suggests that the composites underwent chemical reactions during the sintering process. The possible reactions associated with the generation of TCP and zoisite can be described as [23]:

Ca_10_(PO_4_)_6_(OH)_2_ = 2β-Ca_3_(PO_4_)_2_ + Ca_4_P_2_O_9_+H_2_O↑ (2)

Ca_10_(PO_4_)_6_(OH)_2_ = 3β-Ca_3_(PO_4_)_2_ + CaO+H_2_O↑ (3)

4CaO + 3(3Al_2_O_3_·2SiO_2_) + H_2_O = 2Ca_2_Al_3_(Si_2_O_7_)(SiO_4_)O(OH) + 6Al_2_O_3_ (4)

As shown in Figure 3d, since only a small amount of reaction between CaO and MF occurred, MF kept sharp edges and HA grains were relatively fine. When temperature rose to 1250 °C, the peaks in XRD pattern became broad (Figure 5b—10 wt %), because the content of TCP produced by decomposition of HA was higher. The composites consisted mainly of zoisite and Ca_2_SiO_4_. As stated, the existence of zoisite and Ca_2_SiO_4_ in MF/HA samples forecasts the possibility of sintering reaction during sintering processing. In addition to the reactions at 1150 °C, the formation process of the new phase could be described by the following reaction [24]:

4CaO + 3Al_2_O_3_·2SiO_2_ = 2Ca_2_SiO_4_ + 3Al_2_O_3_ (5)

Furthermore, when the fiber content was increased to 15 wt % (Figure 5b—15 wt %), the composites were substantially completely decomposed to TCP at 1250 °C. Compared with the composites with 10 wt % MF, the increased fiber content promoted more complete decomposition of HA at the same temperature.

When temperature further increased to 1350 °C (Figure 5c—10 wt %), the composites were completely converted into Ca_3_(PO_4_)_2_, accompanied with the formation of kilchoanite (Ca_6_(SiO_4_)(Si_3_O_10_)). The reaction between oxide components (CaO) and 3Al_2_O_3_·2SiO_2_ via the following reaction led to kilchoanite formation along with Al_2_O_3_ [25]:

6CaO + 2(3Al_2_O_3_·2SiO_2_) = Ca_6_(SiO_4_)(Si_3_O_10_) + 6Al_2_O_3_. (6)

The FTIR spectra for as-synthesized MF/HA composites with 10 wt % MF (Figure 5d) strongly support the obtained XRD analysis. Typical absorption bands of functional groups (PO_4_^3−^ and OH^−^) in HA were found. The intense peaks at 569 cm^−1^, 603 cm^−1^, 634 cm^−1^, and 1041 cm^−1^ are ascribed to the major absorption modes of PO_4_^3−^, O-P-O bending mode, and P-O stretching vibration mode, respectively [26]. The peak at 3573 cm^−1^ corresponds to the hydroxyl groups originating from the apatite and zoisite [27,28]. As the temperature increased, the intensity of the peak was significantly reduced and finally disappeared completely. This result is consistent with XRD. Furthermore, the intensified chemical reactions at high temperature led to the production of a large amount of silicate glasses. As seen in Figure 5d, the PO_4_^3−^ peaks at 1041 cm^−1^ and 1089 cm^−1^ appeared to merge. Meanwhile, the absorption peak around 1000 cm^−1^ was extended to the 770–1370 cm^−1^ range, where stretching vibrations of the Si-O-Si and Si-O-Ca bridges occurred [29,30]. Si-O-Si bending vibrations also gave absorption bands at around 500 cm^−1^ [31,32]. This indicates that phosphate tetrahedra were replaced by silicate tetrahedra in the HA structure, and Si could be incorporated into HA in the form of SiO4^4−^ or Si_2_O_7_^6−^ [33,34].

### 3.3. Thermal Analysis

TG–DSC is a remarkable instrument to research the percentage of components, their interactions, and also the structural changes that occur during the heating process, generally known as the degradation of different phases (organic and inorganic). Therefore, TG–DSC was used to further verify the sintering behavior of the composites.

**Figure 6:**
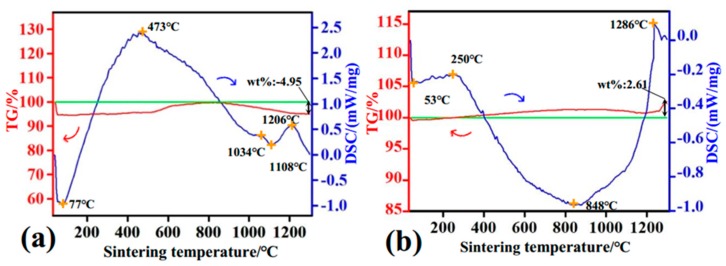
Thermogravimetry–differential scanning calorimetry (TG–DSC) curves of MF/HA composite powders with various MF contents: (**a**) 10 wt %; (**b**) 50 wt %.

Figure 6a shows TG–DSC curve for composites with fiber content of 10 wt %. Based on the analysis of the TG curve, a three-step weight variation was observed. The first weight loss (about 5.24 wt %) was due to the evaporation of adsorbed water [35,36]. This completely corresponds to the first endothermic peak of 77 °C. In the 150–550 °C temperature range, the second weight gain is ascribed to the loss of lattice water owing to the substitution of H_2_O for OH^−^ or HPO_4_^2−^ for PO_4_^3−^ in the apatite lattice, which correlates with the second exothermic peak at 493 °C, as well as the partial dehydroxylation process of HA and the chemical reaction between MF and CaO (from the decomposition of HA) [29]. The degree of reaction was greater than the decomposition rate of HA, resulting in a weight gain of composites at this stage. The third weight loss (about 4.95 wt %) was the result of complete decomposition of HA and the reaction between MF with CaO. The decomposition rate of HA is higher than the reaction rate of MF with CaO at high temperature, thereby reducing the weight. Additionally, when temperature was higher than 813 °C, the DSC curve showed several endothermic peaks and exothermic peaks, indicating that the decomposition of HA and the reaction of MF and CaO are continuous processes. This is consistent with XRD and FTIR results. The effect of MF addition on decomposition of HA was further studied by increasing the fiber content. The thermal stability of composites with 50 wt % addition of MF is reflected in the TG–DSC curve. As can be seen from Figure 6b, two endothermic peaks are present at 53 °C and 848 °C in the DSC curve, corresponding to weight changes in the TG curve. Two exothermic effects occurred at 250 °C and 1286 °C, accompanied with an increase in weight. Meanwhile, there was a considerably broad endothermic peak from 250 °C to 1286 °C. This is because a series of physical and chemical changes occurred in this temperature range (Section 3.2).

### 3.4. Density, Vickers Hardness, and Bending Strength

**Figure 7:**
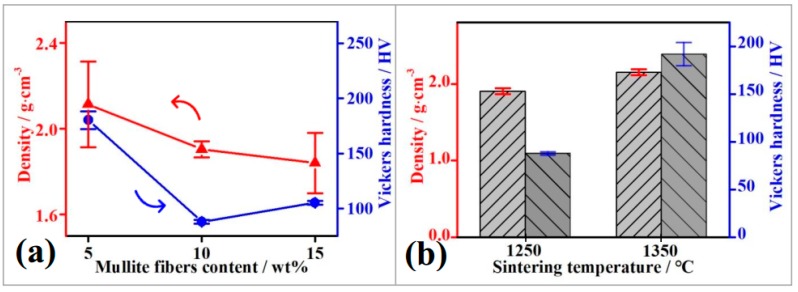
Bulk density and Vickers hardness of MF/HA composites (**a**) with 5 wt %, 10 wt %, and 15 wt % MF addition; (**b**) sintering at 1250 °C and 1350 °C with 10 wt % MF addition.

Figure 7 illustrates the bulk density and Vickers hardness of MF/HA samples with different fiber contents and temperatures. It is clear that bulk density and Vickers hardness curves followed a similar trend. Hence, densification had a direct correlation with Vickers hardness. As shown in Figure 7b, sintered density systematically increased in the temperature range of 1250–1350 °C. At the same time, Vickers hardness increased from 87.93 ± 1.68 HV to 191.87 ± 12.11 HV, and the value of Vickers hardness of the composites sintered at 1350 °C resembled the value of glasses [37]. We hypothesize that these observations can mostly be attributed to densification, micro-pores elimination, and reaction fusion. These results are consistent with those of SEM and XRD which, also showed an extended formation of silicate glasses. In contrast, a diverse behavior for the composites was observed by increasing the fiber content (Figure 7a). Both the sintered density and Vickers hardness decreased with increasing fiber content. Comparing the Vickers hardness data, it can be said that the Vickers hardness values of 180.23 ± 8.16 HV, 87.93 ± 1.68 HV, and 105.20 ± 1.65 HV for 5 wt %, 10 wt %, and 15 wt % MF additions respectively were superior to the recently achieved Vickers hardness of HA composites [38].

Table 1 shows the relative density values of MF/HA composites at different fiber contents and sintering temperatures. It can be seen that the relative density of MF/HA composites decreased with increasing fiber content. This is because a fiber agglomeration phenomenon occurred in the composites. However, the relative density of MF/HA composites increased with increasing sintering temperature. This can be explained by the fact that an increase in the sintering temperature can promote the fusion of the HA grains and the extensive reaction of the composites. Thence, the relative density of the composites increased and reached a maximum value of 68.05% at 1350 °C.

In this study, one of the main focuses is the influence of sintering temperature and fiber content on the variation of density and Vickers hardness of MF/HA composites. The influence of sintering temperature and fiber content on sintering behavior of the composites can be described in terms of the following two aspects. With increasing fiber content, mullite–mullite contacts in the composites may act as weak interfaces, and the composites containing higher MF fractions are tend more towards the decomposition of HA to form a lower-density phase (TCP). Thereafter, MF reacts with CaO to form a calcium-alumino-silicate phase and the reactions are favorable for the decomposition of HA. The decomposition of HA generates softer sintered phases such as CaO and silicate [39,40]. With increasing sintering temperature, the reaction between MF and CaO yields a large amount of silicate glasses, which increases the driving force of particle rearrangement. The more liquid phase produced, the lower the viscosity of the composites, which increases the mass transfer in liquid phase, sintering driving force, and grain boundary movement rate. The densification of the samples increases and porosity of the samples decreases. Accordingly, bulk density and Vickers hardness increase as sintering temperature increases.

**Figure 8:**
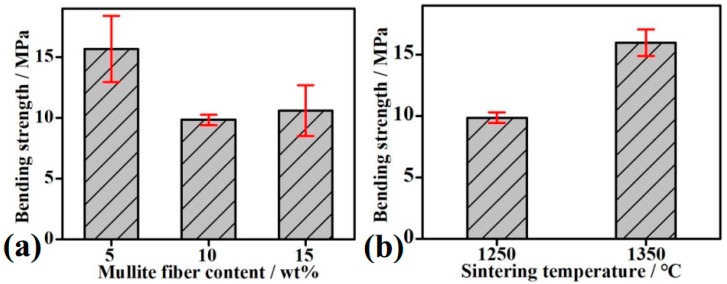
Bending strength of MF/HA composites: (**a**) with 5 wt %, 10 wt %, and 15 wt % MF addition; (**b**) sintering at 1250 °C and 1350 °C with 10 wt % MF addition.

The bending strength of MF/HA composites are evaluated in Figure 8. As shown in Figure 8a, when the fiber content was 5 wt %, bending strength showed a relatively high value of 15.69 ± 2.73 MPa, which is slightly higher than that of pure HA in a previous study [41]. When fiber content was 10 wt %, the composites underwent extensive chemical reactions during the sintering process. The decomposition rate of HA became much faster. It is therefore assumed that the more CaO is decomposed from HA, the more fibers can be corroded and damaged. Meanwhile, the formation of glass phases (from reaction between MF and CaO) is accelerated. The glass phases accumulate at grain boundaries. Accumulation of glass phases on grain boundaries and weak link of fibers result in a lower grain bond strength and bending strength of composites. When the fiber content was increased to 15 wt %, the bending strength increased slightly. This is because the further addition of fibers also accelerated the decomposition of HA, which promoted the rearrangement of particles and the filling of pores. Therefore, the composites with 15 wt % MF had a slightly higher bending strength. On the other hand, the bending strength of composites at 1350 °C (15.96 ± 1.09 MPa) was 1.62 times higher than that at 1250 °C (9.86 ± 0.43 MPa) in Figure 8b. This is because the fusion effect between MF and matrix was significantly improved. Simultaneously, both mass transfer across/along grain boundaries and density increased at high temperature.

**Figure 9:**
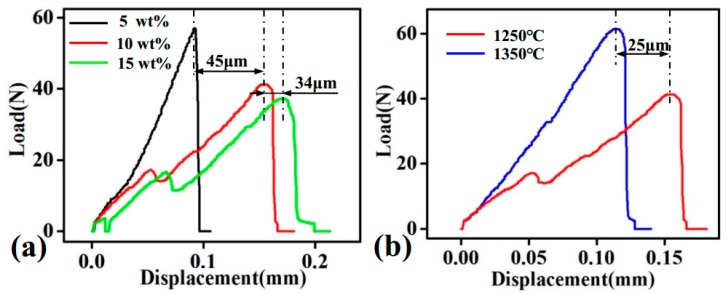
Load versus displacement plot of the composites: (**a**) sintering at 1250 °C with 5 wt %, 10 wt %, and 15 wt % MF addition; (**b**) sintering at 1250 °C and 1350 °C with 10 wt % MF addition.

Figure 9 presents the load versus displacement curve for the representative MF/HA composites. It can be seen that the slope of the curve was relatively high. After a relatively short displacement, the load reached a maximum and then fell rapidly (Figure 9a). This indicates that composites showed obvious brittle fracture behavior [42]. As fiber content increased, sample fracture occurred after a larger displacement, meaning that the pseudoplasticity of the composites improved [43]. This pseudoplasticity is attributed to the good bonding of MF to the HA matrix. Meanwhile, abundant fracture energy was exhausted by fibers which did not participate in the reaction. This indicates that the content of MF had a significant influence on the bending strength and fracture behavior of the composites. In addition, the composites obtained the maximum load with increased sintering temperature. However, after the load reached a maximum value, it dropped off rapidly. The samples underwent the overall fracture after the smaller displacement (Figure 9b), which was responsible for the brittle failure within the samples.

The densification mechanism had a significant impact on the properties of MF/HA composites due to different sintering temperatures and the addition of MF. As described later, when sintering temperature was raised to 1150 °C, MF was restricted to be in contact with several surrounding HA particles. As holding time progressed, grains were generated by activating mass transfer mechanisms and interdiffusion of the primary particles. HA was partially dehydroxylated to produce CaO, which would enable MF to react with CaO. When sintering at a temperature of 1250 °C, densification behavior occurred by particle rearrangement and the number of pores started to decrease. Moreover, the combination of grains triggered the formation of sintering necks, causing the development of a vermicular structure. When the temperature rose to 1350 °C, densification was limited by the incomplete disappearance of pores, with a small number of closed pores retained between the grains. Moreover, grain growth and merger through dissolving small grains were observable. Therefore, sintering at higher temperature made the composites achieve densification and greatly enhanced their mechanical properties [44]. In contrast, the further addition of MF had a negative impact on densification and mechanical properties of MF/HA composites. MF promotes the decomposition of HA to form CaO at high temperature. Then, the alkaline earth oxide CaO breaks or weakens Si-O-Si bridges and Al-O-Al linkages in MF and hence produces a decline of silica’s three-dimensional network construction. Thus, the metal ions in Al_2_O_3_ and SiO_2_ are replaced by calcium ions and structural degradation of samples occurs [45]. Thereafter, further addition of fibers cannot achieve the desired enhancement effect due to the destruction of fiber integrity and the loss of their excellent properties. In addition, although the etching rate of Ca ions at room temperature is so small as to be inappreciable, the reaction rate of the composites sintered at 1150–1350 °C can be a few orders of magnitude higher than that at room temperature. This is because the rate of chemical reaction follows the Arrhenius equation [46,47]. Therefore, this phenomenon not only limits complete densification but also reduces mechanical properties such as bending strength. One can speculate that fiber content and sintering temperature have different effects on the sintering behavior of the composites.

## 4. Conclusions

This paper presents results on microstructure details, phase assemblage, chemical reactions, densification mechanism, and properties of MF/HA composites. The following major conclusions can be drawn. As MF content increased, the flat morphology of composites transferred into a rugged structure. Meanwhile, the further addition of fiber reduced the thermal stability of HA, resulting in a slight decrease in HA decomposition temperature and forming a large amount of CaO. Alkaline earth oxide CaO subsequently could break or weaken Si-O-Si bridges and Al-O-Al linkages of MF. The further addition of fiber resulted in a faster rate of sintering reaction and the formation of many silicate glasses. The strength index of the composites showed a downward trend. Compared with the brittle fracture behavior of low-fiber composites, the high-fiber samples displayed overall fracture after a larger displacement. This is because abundant of fracture energy was exhausted by fibers which did not participate in reaction. When sintering temperature increased from 1150 °C to 1350 °C, the decomposition rate of HA increased. Bulk density and Vickers hardness of the composites sintered at 1350 °C for fiber additions of 10 wt % reached to 2.153 g/cm^3^ and 191.87 ± 12.11 HV, respectively. This was mainly due to the densification of organizational structure and the formation of silicate glasses during the sintering process. The composite densification process included grain boundary diffusion, grain rearrangement, grain growth and merger, until the disappearance of a large number of pores. A series of chemical reactions occurred, with the generation of various products such as zoisite or kilchoanite. The study is expected to open new insights on the properties of using mullite to reinforce calcium phosphate salt.

## Figures and Tables

**Table 1:** Relative density of MF/HA composites.

Performance Parameters	Fiber Content (wt %)–Sintering Temperature (°C)			
	5–1250	10–1250	15–1250	10–350
Theoretical density (g/cm^3^)	3.1648	3.1645	3.1643	3.1645
Sintered density (g/cm^3^)	2.1134	1.9040	1.8400	2.1534
Relative density (%)	66.78	60.17	58.15	68.05

## References

[1] Koutsopoulos S. (2002). Synthesis and characterization of hydroxyapatite crystals: A review study on the analytical methods. J. Biomed. Mater. Res..

[2] Samavedi S., Whittington A.R., Goldstein A.S. (2013). Calcium phosphate ceramics in bone tissue engineering: A review of properties and their influence on cell behavior. Acta Biomater..

[3] Koutsopoulos S. (2001). Kinetic Study on the Crystal Growth of Hydroxyapatite. Langmuir.

[4] Zhang X., Chang W., Lee P., Wang Y., Yang M., Li J., Kumbar S., Yu X. (2014). Polymer-Ceramic Spiral Structured Scaffolds for Bone Tissue Engineering: Effect of Hydroxyapatite Composition on Human Fetal Osteoblasts. PLoS ONE.

[5] Fidancevska E., Ruseska G., Bossert J., Lin Y.M., Boccaccini A.R. (2007). Fabrication and characterization of porous bioceramic composites based on hydroxyapatite and titania. Mater. Chem. Phys..

[6] Reilly D.T., Burstein A.H. (1976). The Mechanical Properties of Cortical Bone. JBJS.

[7] Ravikumar K., Mallik P.K., Basu B. (2016). Twinning induced enhancement of fracture toughness in ultrafine grained Hydroxyapatite-Calcium Titanate composites. J. Eur. Ceram. Soc..

[8] Suchanek W., Yoshimura M. (1998). Processing and properties of hydroxyapatite-based biomaterials for use as hard tissue replacement implants. J. Mater. Res..

[9] Viswanath B., Ravishankar N. (2006). Interfacial reactions in hydroxyapatite/alumina nanocomposites. Scr. Mater..

[10] Radha G., Balakumar S., Venkatesan B., Vellaichamy E. (2015). Evaluation of hemocompatibility and in vitro immersion on microwave-assisted hydroxyapatite-alumina nanocomposites. Mater. Sci. Eng. C.

[11] Gautam C.R., Tamuk M., Manpoong C.W., Gautam S.S., Kumar S., Singh A.K., Mishra V.K. (2016). Microwave synthesis of hydroxyapatite bioceramic and tribological studies of its composites with SrCO_3_ and ZrO_2_. J. Mater. Sci..

[12] Ponta O., Ciceo-Lucacel R., Vulpoi A., Radu T., Simon V., Simon S. (2015). Synthesis and characterisation of nanostructured silica-powellite-HAP composites. J. Mater. Sci..

[13] Zhu M., Zhang J., Zhao S., Zhu Y. (2016). Three-dimensional printing of cerium-incorporated mesoporous calcium-silicate scaffolds for bone repair. J. Mater. Sci..

[14] Sadik C., Amrani I.E.E., Albizane A. (2014). Recent advances in silica-alumina refractory: A review. J. Asian Ceram. Soc..

[15] Duval D.J., Risbud S.H., Shackelford J.F., Shackelford J.F., Doremus R.H. (2008). Mullite Structure, Properties and Processing. Ceramic and Glass Materials.

[16] Anggono J. (2005). Mullite Ceramics: Its Properties, Structure, and Synthesis. J. Tek. Mesin.

[17] Dubey A.K., Sitesh G., Nath S., Basu B. (2011). Spark plasma sintering to restrict sintering reactions and enhance properties of hydroxyapatite-mullite biocomposites. Ceram. Int..

[18] Ebadzadeh T., Behnamghader A., Nemati R. (2011). Preparation of porous hydroxyapatite ceramics containing mullite by reaction sintering of clay, alumina and hydroxyapatite. Ceram. Int..

[19] Yetmez M., Erkmen Z.E., Kalkandelen C., Ficai A., Oktar F.N. (2017). Sintering effects of mullite-doping on mechanical properties of bovine hydroxyapatite. Mater. Sci. Eng. C.

[20] Nath S., Biswas K., Wang K., Bordia R.K., Basu B. (2010). Sintering, Phase Stability, and Properties of Calcium Phosphate-Mullite Composites. J. Am. Ceram. Soc..

[21] Nath S., Biswas K., Basu B. (2008). Phase stability and microstructure development in hydroxyapatite-mullite system. Scr. Mater..

[22] Cordell J.M., Vogl M.L., Johnson A.J.W. (2009). The influence of micropore size on the mechanical properties of bulk hydroxyapatite and hydroxyapatite scaffolds. J. Mech. Behav. Biomed. Mater..

[23] Muralithran G., Ramesh S. (2000). The effects of sintering temperature on the properties of hydroxyapatite. Ceram. Int..

[24] Shen M., Qiu K., Zhang L., Huang Z., Wang Z., Liu J. (2015). Influence of Coal Blending on Ash Fusibility in Reducing Atmosphere. Energies.

[25] Huggins F.E., Kosmack D.A., Huffman G.P. (1981). Correlation between ash-fusion temperatures and ternary equilibrium phase diagrams. Fuel.

[26] Blakeslee K.C., Condrate R.A. (1971). Vibrational Spectra of Hydrothermally Prepared Hydroxyapatites. J. Am. Ceram. Soc..

[27] Weis F.A., Lazor P., Skogby H., Stalder R., Eriksson L. (2016). Polarized IR and Raman spectra of zoisite: Insights into OH-dipole orientation and the luminescence. Eur. J. Miner..

[28] Legeros R.Z., Bonel G., Legros R. (1978). Types of “H_2_O” in human enamel and in precipitated apatites. Calcif. Tissue Int..

[29] Karakassides M.A., Gournis D., Petridis D. (1999). An infrared reflectance study of Si-O vibrations in thermally treated alkali-saturated montmorillonites. Clay Miner..

[30] Frost R.L., Palmer S.J., Reddy B.J. (2007). Near-infrared and mid-IR spectroscopy of selected humite minerals. Vib. Spectrosc..

[31] Marchi J., Morais D.S., Schneider J., Bressiani J.C., Bressiani A.H.A. (2005). Characterization of rare earth aluminosilicate glasses. J. Non-Cryst. Solids.

[32] Veres R., Vanea E., Gruian C., Baia L., Simon V. (2014). The effects of peg assisted synthesis and zinc addition on gamma irradiated bioactive glasses. Compos. Part B.

[33] Li K., Guo Q., Zhang L., Zhang Y., Liu S., Guo K., Li S. (2017). Synthesis and characterization of Si-substituted hydroxyapatite bioactive coating for SiC-coated carbon/carbon composites. Ceram. Int..

[34] Tang X.L., Xiao X.F., Liu R.F. (2005). Structural characterization of silicon-substituted hydroxyapatite synthesized by a hydrothermal method. Mater. Lett..

[35] Bai J. (2010). Fabrication and properties of porous mullite ceramics from calcined carbonaceous kaolin and α-Al_2_O_3_. Ceram. Int..

[36] Chen Y.F., Wang M.C., Hon M.H. (2004). Kinetics of secondary mullite formation in kaolin-Al_2_O_3_ ceramics. Scr. Mater..

[37] Agathopoulos S., Nikolopoulos P., Salomoni A., Tucci A., Stamenkovic I. (1996). Preparation and properties of binary oxide bioceramics. J. Mater. Sci. Mater. Med..

[38] Oktar F.N., Agathopoulos S., Ozyegin L.S., Gunduz O., Demirkol N., Bozkurt Y., Salman S. (2007). Mechanical properties of bovine hydroxyapatite (BHA) composites doped with SiO_2_, MgO, Al_2_O_3_, and ZrO_2_. J. Mater. Sci. Mater. Med..

[39] Nath S., Dey A., Mukhopadhyay A.K., Basu B. (2009). Nanoindentation response of novel hydroxyapatite-mullite composites. Mater. Sci. Eng. A.

[40] Witek S.R., Miller G.A., Harmer M.P. (2010). Effects of CaO on the Strength and Toughness of AIN. J. Am. Ceram. Soc..

[41] Zhao X., Wang X., Xin H., Zhang L., Yang J., Jiang G. (2018). Controllable preparation of SiC coating protecting carbon fiber from oxidation damage during sintering process and SiC coated carbon fiber reinforced hydroxyapatite composites. Appl. Surf. Sci..

[42] Murakami S., Kato K., Enari Y., Kamitakahara M., Watanabe N., Ioku K. (2012). Hydrothermal synthesis of porous hydroxyapatite ceramics composed of rod-shaped particles and evaluation of their fracture behavior. Ceram. Int..

[43] Yan S., He P., Jia D., Yang Z., Duan X., Wang S., Zhou Y. (2016). Effect of fiber content on the microstructure and mechanical properties of carbon fiber felt reinforced geopolymer composites. Ceram. Int..

[44] He Z., Ma J., Wang C. (2005). Constitutive modeling of the densification and the grain growth of hydroxyapatite ceramics. Biomaterials.

[45] Chrissanthopoulos A., Bouropoulos N., Yannopoulos S.N. (2008). Vibrational spectroscopic and computational studies of sol-gel derived CaO-MgO-SiO_2_ binary and ternary bioactive glasses. Vib. Spectrosc..

[46] Kikuyama H. (1994). A Study of the Dissociation State and the SiO_2_ Etching Reaction for HF Solutions of Extremely Low Concentration. J. Electrochem. Soc..

[47] Laidler K.J. (1984). The development of the Arrhenius equation. J. Chem. Educ..

